# Evaluation of community-based screening tools for the early screening of osteoporosis in postmenopausal Vietnamese women

**DOI:** 10.1371/journal.pone.0266452

**Published:** 2022-04-05

**Authors:** My Hanh Bui, Phuoc Thang Dao, Quynh Long Khuong, Phuong-Anh Le, Thuy-Trang Thi Nguyen, Gia Du Hoang, Tu Hoang Le, Hoai Thu Pham, Hong-Xuyen Thi Hoang, Quang Cuong Le, Xuan Thanh Dao

**Affiliations:** 1 Hanoi Medical University, Hanoi, Vietnam; 2 Hanoi Medical University Hospital, Hanoi, Vietnam; 3 Interactive Research and Development, Ho Chi Minh City, Vietnam; 4 Hanoi University of Public Health, Hanoi, Vietnam; 5 Bach Mai Hospital, Dong Da, Hanoi, Vietnam; 6 Viet Duc Hospital, Hoan Kiem, Hanoi, Vietnam; University of Brescia: Universita degli Studi di Brescia, ITALY

## Abstract

**Background:**

Osteoporosis affects people worldwide. However, there are few validated tools for the early screening of osteoporosis in Vietnam. We set out to evaluate the performance of the osteoporosis self-assessment tool for Asians (OSTA) and the osteoporosis screening tool for Chinese (OSTC) for the early screening of osteoporosis in postmenopausal Vietnamese women.

**Methods:**

We analyzed retrospective data from 797 postmenopausal Vietnamese women. The bone mineral density (BMD) in the lumbar vertebrae (L1–L4) and the left and right femoral necks of all participants were measured using dual-energy X-ray absorptiometry (DXA). Osteoporosis was defined as the BMD (T-score) < -2.5. The OSTA and OSTC scores were calculated from the age and weight of participants. Receiver operating characteristic analysis was conducted to compare the performance of the two tools with the BMD measurements by DXA at different anatomical sites.

**Results:**

The rates of osteoporosis determined by BMD varied between anatomical sites, and ranged from 43.4% to 54.7% in the lumbar vertebrae and 29.2% and 8.9% in the left and right femoral necks, respectively. For the vertebrae, the area under the curve (AUC) for OSTA ranged from 70.9% to 73.9% and for OSTC ranged from 68.7% to 71.6%. The predictive value of both tools was higher for femoral necks, with the AUC of OSTA for the left and right femoral necks being 80.0% and 85.8%, respectively. The corresponding figures for OSTC were 80.5% and 86.4%, respectively. The highest sensitivity and specificity of OSTA were 74.6% and 81.4%, while these figures for OSTC were 73.9% and 82.6%, respectively.

**Conclusion:**

OSTA and OSTC were shown to be useful self-assessment tools for osteoporosis detection in Vietnam postmenopausal women. Further research is encouraged to determine the applicability of tools for other populations and settings.

## Introduction

Osteoporosis is a metabolic disorder that causes bone mass reduction, leading to macro- and micro-destruction of different parts of the human skeleton [1]. Skeletal micro-abnormalities progressively reduce bone sustainability and result in a risk of fragility fracture that can take the lives of those affected [1,2]. Osteoporotic fracture affects people globally and has gradually increased in significance to become a considerable public health issue [3]. The European Union (EU) reported that 22 million women and 5.5 million men were diagnosed with osteoporosis in EU countries according to World Health Organization (WHO) criteria in 2010 [4]. The report also showed that most of the fracture cases occurred in women, of whom 3.3 million over the age of 50 suffered hip fractures [4] Baccaro et al. [5] found that the percentage of women with osteoporosis in Brazil varied between 15% and 33%. The figure for American women over 50 years old was 15.4% in 2010, and the rate increased with age, with the highest rate in the over-65s [5]. Kanis et al. [4] estimated that the prevalence of fragility fractures would increase by 28% in the EU by 2025; a similar trend is expected for Italy over the next 25 years [1]. The China Osteoporosis Prevalence study conducted in 2018 found that the prevalence of osteoporosis in China was 20.6% and 5% for women and men over 40 years old, respectively [6]. Yeap et al. found in data recorded between 2016 and 2018 that 14% of the Malaysian population over 45 years old had osteoporosis [7].

Osteoporosis-related fractures cause not only loss of self-sufficiency but also increase the risk of disability and mortality, with a mortality rate of 9% and 36% one month and one year after fracture events, respectively [1]. Along with severe outcomes, patients with fragility fractures face the risk of institutionalization that consequently results in a loss of quality-adjusted life-years (QALY), feelings of isolation, and mental disorders relating to depression [1]. An osteoporotic fracture requires long-term treatment and specific medical care, incurring significant pharmacological costs [8,9].

The risk of osteoporosis increases with age [1–3], and has a noticeably greater impact on females than males due to age-linked hormonal imbalances, especially during the postmenopausal period [1,4,10]. Osteoporosis-related postmenopausal fractures commonly occur in the vertebrae, femur, hip, and distal forearm, potentially leading to disability and mortality [1–3]. The WHO suggests using BMD as the diagnostic tool for osteoporosis, measured using DXA.

Vietnam is a low-middle income country (LMIC) located in Southeast Asia with a population of 96.5 million [11]. GDP per capita was 2,715 USD in 2019 [12]. The prevalence of osteoporosis in Vietnam varies from 14% to 16% between studies [13–15] and is increasing [14]. In line with global population aging, the proportion of over-50s in Vietnam has risen over the past decade and this is expected to increase further the prevalence of osteoporosis. This issue highlights the need for rapid and cost-effective screening tools for osteoporosis to overcome the limitations of DXA in the context of community-based screening. Although many screening tools for predicting osteoporosis and fracture risk have been published and show promising performance, with sensitivities up to 91% [16–23], local differences mean that there are challenges in using these tools in different nations.

The osteoporosis self-assessment tool for Asians (OSTA) and the osteoporosis screening tool for Chinese (OSTC) have been developed and evaluated in Asia countries [16,24,25]. These are simple tools, using only weight and age as predictors, and they are appropriate in the context of community-based screening and low-resource settings. However, OSTA and OSTC have not been validated specifically among postmenopausal Vietnamese women. This limitation necessitates research to explore the use of OSTA and OSTC in this high-risk group. Our study was conducted to evaluate the performance of OSTA and OSTC in osteoporosis prediction among postmenopausal Vietnamese women.

## Materials and methods

### Data source and participants

We used retrospective data on women over 18 years of age who had visited the Department of Functional Exploration at Hanoi Medical University Hospital, Vietnam. The data were extracted from the health information system (HIS), from July 1, 2018, to January 2, 2021. The selection criteria were: (1) postmenopausal women without any anatomical abnormalities of the neck of the femur or lumbar vertebrae; (2) who had not undergone spinal or femur surgery; and (3) who did not have risk factors for secondary osteoporosis, including type 1 diabetes mellitus, long-term corticosteroid therapy, or rheumatoid arthritis. The final sample size was 797 participants. The participants’ characteristics, including age, history of medical diagnosis, age at the start of menopause, and pharmaceutical therapy, were collected at the time patients were registered by specialists.

### Measurements

#### Body mass index

Anthropometric measurements (height and weight) were obtained by specialists using standard procedures [26]. Weight and height were measured twice with light clothing and without shoes using a Tanita WB-380H digital scale. The average of the measurements was used in the analysis. The body mass index (BMI, kg/m^2^) was calculated by weight (kg)/height^2^ (m) and was then categorized according to the criteria for Asian populations [27].

#### Bone mineral density

The BMD in the lumbar vertebrae (L1–L4) and the left and right femoral neck of all participants were measured using a Discovery Ci DXA system (Hologic, USA, 2019). For Asians, this DXA machine uses the reference parameters of the Japanese female population [28]. DXA measures BMD with X-ray energy from two sources passing through bone tissue [29]. The BMD value is then converted to a *T*-score, which is represented by the standard deviation of the mean bone mass peak [1]. According to the WHO, a *T*-score ≥ -1.0 indicates a normal BMD, between -1.0 and -2.5 indicates osteopenia (low BMD), and *T*-score ≤ -2.5 indicates osteoporosis [1].

#### OSTA

The OSTA score was calculated using the formula: [weight (kg)—age (year)] x 0.2. A lower score indicates a higher risk of bone fracture. The OSTA score was then classified into three categories: OSTA score > -1 indicates a low risk of bone fracture, OSTA score between -4 and -1 indicates a medium risk of bone fracture, and < -4 indicates a high risk of bone fracture [30].

#### OSTC

The OSTC score indicates the risk of osteoporosis and was calculated using the formula: weight (kg)–(2 x age (year)) + 50, with a lower score indicating a higher risk of osteoporosis. OSTC score > 0 indicates no risk of osteoporosis, while OSTC ≤ 0 indicates an individual is at risk of osteoporosis [17].

### Statistical analyses

We used frequencies and percentages to describe categorical variables, and means and standard deviations to describe quantitative variables. Receiver operating characteristic (ROC) analysis was conducted to evaluate the predictive value of OSTA and the OSTC. The AUC of OSTA and OSTC in diagnosing osteoporosis were identified based on the BMD *T*-score value. AUC < 70% indicates poor classification accuracy, AUC between 70% and 80% indicates medium classification accuracy, and AUC ≥ 80% indicates good classification accuracy [31]. The Youden index (*Sensitivity* + *Specificity*– 1) was used to determine the optimal cut-off points for identifying the sensitivity and specificity of the OSTA and OSTC. All analyses were conducted using R 4.0.0 (R Foundation for Statistical Computing, Vienna, Austria). The type I error rate was set at 0.05.

### Ethical considerations

We used retrospective and anonymized data for this non-interventional study. Therefore, the ethics approval was waived. The data were obtained with permission from the Hanoi Medical University Hospital Research Review Board.

## Results

Table 1 describes the characteristics of the participants. The mean ± SD age and age at the start of menopause of the participants were 64.1 ± 7.7 and 49.8 ± 3.1, respectively. Nearly half of the women started menopause at the age of 50 to 54 (42.9%). The mean weight and height of participants were 52.5 ± 9.1 kg and 150.0 ± 9.7 cm, respectively. Half of the participants were overweight or obese (BMI ≥ 23 kg/m^2^).

**Table 1:** Demographic characteristics (N = 797).

Characteristic	n (%)/ Mean ± SD
**Age** (years), *mean ± SD*	64.1 ± 7.7
**Age group**, *n (%)*	
< 55 y	60 (7.5)
55–59	203 (25.5)
60–64	189 (23.7)
65–69	140 (17.6)
70–74	123 (15.4)
≥ 75	82 (10.3)
**Menopause age** (years), *mean ± SD*	49.8 ± 3.1
**Menopause age group**, *n (%)*	
45–49	327 (35.3)
50–54	397 (42.9)
≥ 55	73 (7.9)
**Height** (cm), *mean ± SD*	150.0 ± 9.7
**Weight** (kg), *mean ± SD*	52.5 ± 9.1
**BMI** (kg/m^2^), *mean ± SD*	23.0 ± 2.9
**BMI group**, *n (%)*	
< 18.5 kg/m^2^	50 (6.3)
18.5–22.9 kg/m^2^	347 (43.8)
23.0–24.9 kg/m^2^	219 (27.6)
≥ 25 kg/m^2^	177 (22.3)

BMI: Body mass index, SD: Standard deviation.

Table 2 shows the OSTA and OSTC scores and T-scores of different anatomical sites. The mean OSTA and OSTC scores were -2.3 ± 2.5 and -25.7 ± 18.9, respectively. The mean T-scores of the lumbar vertebrae (L1 to L4) were similar and ranged from -2.5 to -2.2. This score was higher in the left and right femoral neck, at -1.9 and -1.2, respectively. The OSTA and OSTC scores and T-scores were lower among older groups and participants with a BMI < 18.5 kg/m^2^.

**Table 2:** Distribution of scores for the two screening tools and BMD (T-score) by age and BMI groups.

Characteristic	OSTA	OSTC	T-score						
			Lumbar vertebra L1	Lumbar vertebra L2	Lumbar vertebra L3	Lumbar vertebra L4	Lumbar vertebrae (L1–L4)	Left femoral neck	Right femoral neck
**Overall**	-2.3 ± 2.5	-25.7 ± 18.9	-2.3 ± 1.0	-2.4 ± 1.2	-2.5 ± 1.3	-2.2 ± 1.4	-2.4 ± 1.2	-1.9 ± 1.0	-1.2 ± 1.0
**Age** (year)									
< 55	0.0 ± 1.5	-1.9 ± 8.0	-1.6 ± 0.9	-1.8 ± 1.0	-1.8 ± 1.2	-1.5 ± 1.5	-1.7 ± 1.1	-1.3 ± 0.9	-0.5 ± 0.8
55–59	-0.7 ± 1.4	-10.6 ± 7.7	-2.1 ± 1.0	-2.1 ± 1.2	-2.2 ± 1.3	-1.9 ± 1.3	-2.1 ± 1.1	-1.3 ± 0.9	-0.6 ± 0.9
60–64	-1.7 ± 2.2	-20.3 ± 11.2	-2.3 ± 1.0	-2.3 ± 1.1	-2.4 ± 1.2	-2.1 ± 1.2	-2.3 ± 1.0	-1.9 ± 0.8	-1.1 ± 0.8
65–69	-3.1 ± 1.5	-32.4 ± 7.8	-2.5 ± 1.0	-2.6 ± 1.1	-2.8 ± 1.3	-2.4 ± 1.4	-2.6 ± 1.1	-2.1 ± 1.0	-1.3 ± 0.9
70–74	-4.0 ± 2.4	-41.3 ± 12.4	-2.7 ± 0.9	-2.7 ± 1.2	-3.0 ± 1.4	-2.6 ± 1.4	-2.8 ± 1.1	-2.5 ± 0.8	-1.7 ± 0.9
≥ 75	-5.8 ± 1.9	-57.9 ± 10.9	-2.7 ± 1.0	-2.9 ± 1.2	-3.1 ± 1.4	-2.6 ± 1.5	-2.8 ± 1.2	-2.8 ± 0.9	-2.1 ± 0.9
**BMI** (kg/m^2^)									
< 18.5	-5.5 ± 2.0	-44.0 ± 18.1	-3.1 ± 0.8	-3.3 ± 1.2	-3.7 ± 1.4	-3.5 ± 1.4	-3.4 ± 1.1	-2.8 ± 0.8	-2.1 ± 0.9
18.5–22.9	-3.0 ± 2.0	-28.7 ± 16.9	-2.5 ± 1.0	-2.5 ± 1.1	-2.7 ± 1.2	-2.3 ± 1.2	-2.5 ± 1.1	-2.0 ± 0.9	-1.3 ± 0.9
23.0–24.9	-1.8 ± 1.9	-22.2 ± 16.7	-2.2 ± 1.0	-2.2 ± 1.1	-2.3 ± 1.3	-2.0 ± 1.3	-2.2 ± 1.1	-1.8 ± 1.0	-1.0 ± 1.0
≥ 25	-0.9 ± 2.2	-19.7 ± 18.4	-2.0 ± 1.0	-2.0 ± 1.2	-2.1 ± 1.3	-1.7 ± 1.5	-1.9 ± 1.2	-1.7 ± 1.1	-0.9 ± 1.0

BMI: Body mass index; OSTA: Osteoporosis self-assessment tool for Asians; OSTC: Osteoporosis screening tool for Chinese.

Data presented as mean ± SD.

**Fig 1:**
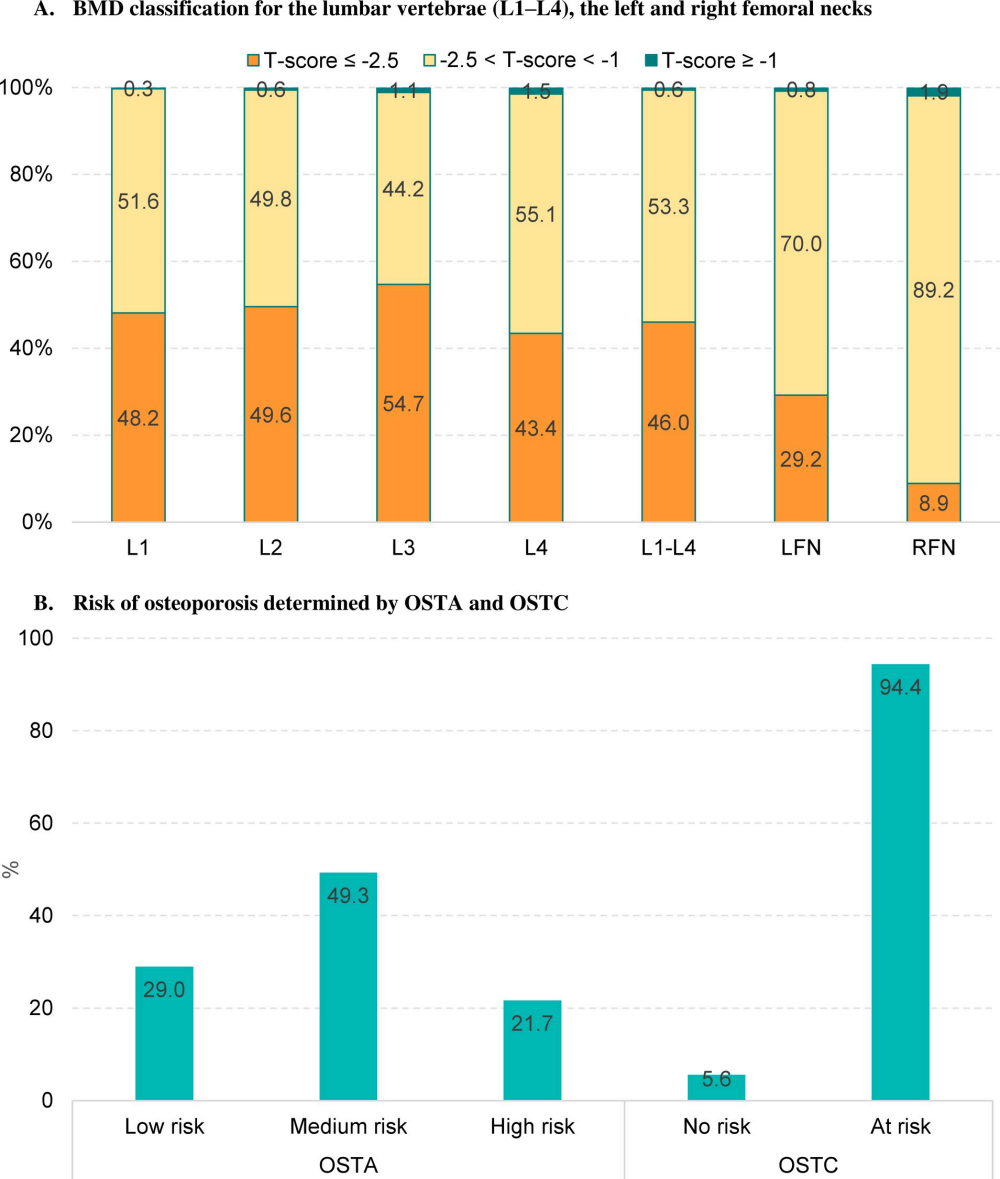
BMD classification and risk of osteoporosis determined by OSTA and OSTC.

Fig 1 presents the BMD classification, risk of fracture, and risk of osteoporosis determined by OSTA and OSTC. The prevalence of osteoporosis (T-score ≤ -2.5) detected in lumbar vertebrae L1 to L4 ranged from 43.4% to 54.7% and was highest at lumbar vertebra L3 and lowest at lumbar vertebra L4. For the left and right femoral necks, 29.2% and 8.9% of the participants were classified as having osteoporosis, respectively. OSTA predicted that half of the women were at medium risk and 21.7% were at high risk of fracture. According to OSTC, almost all the participants were at risk of osteoporosis (94.4%). The detail on the comparison between the risk of osteoporosis determined by OSTA and OSTC and the BMD classification in different anatomical sites is shown in S1 Table.

**Fig 2:**
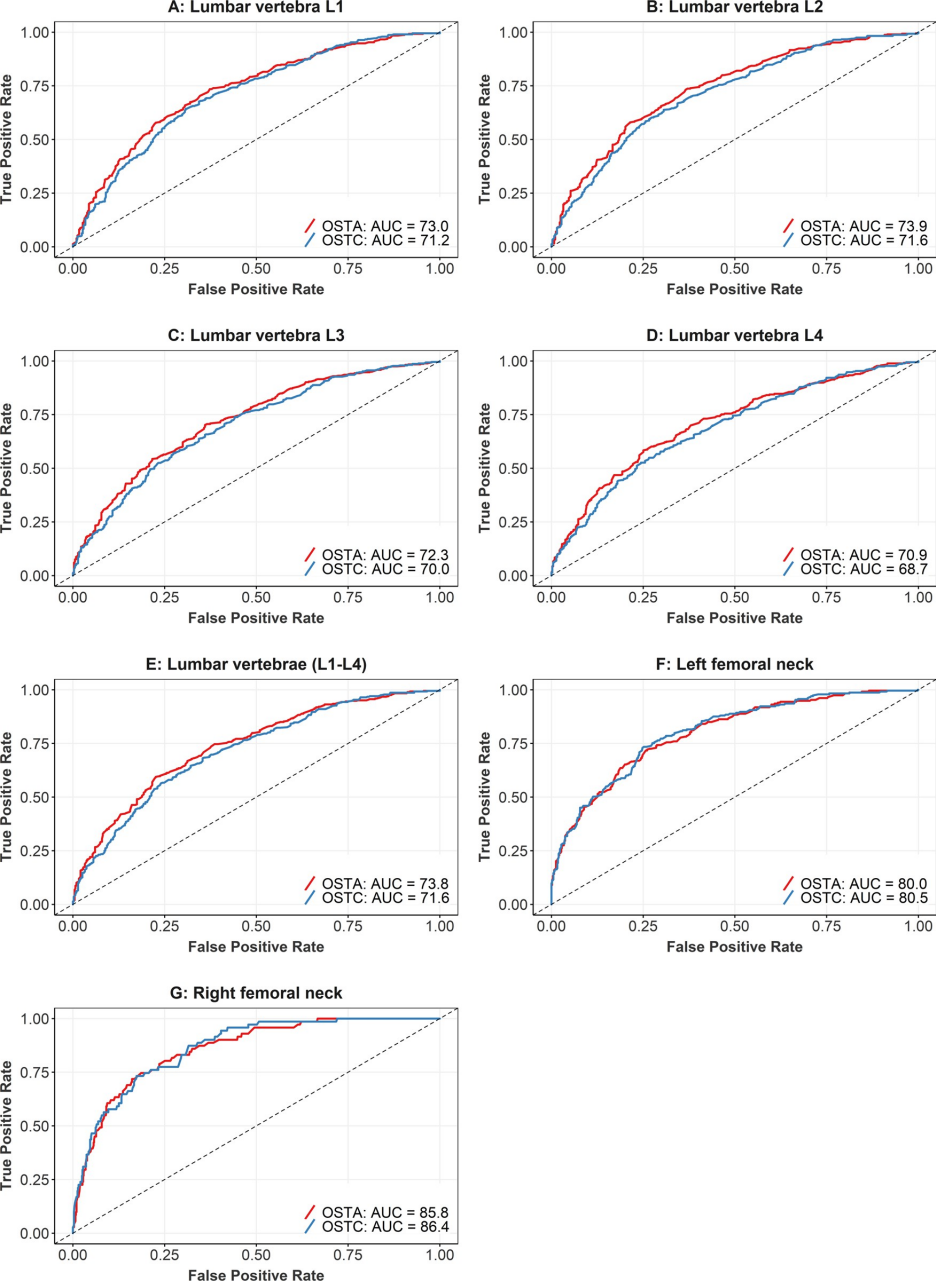
ROC analysis of the predictive value of the OSTA and OSTC as compared to the BMD measurement in different anatomical sites.

Table 3 and Fig 2 show the predictive validity of OSTA and OSTC for different anatomical sites. For the vertebrae, the AUC values of OSTA ranged from 70.9% to 73.9%, with a minimum lower boundary of confidence intervals of 67.3%. The corresponding optimal cut-offs of OSTA were chosen at -1.93, -1.89, -1.93, and -2.93 for lumbar vertebrae L1 to L4 respectively, which generated sensitivity from 58.4% to 73.7% and specificity from 62.7% to 74.9%. Similar results were found for OSTC, where the AUC value for OSTC in predicting osteoporosis in the vertebrae varied from 68.7% to 71.6%, with sensitivity from 54.9% to 73.9% and specificity from 56.0% to 73.4%.

The predictive value of both tools was higher for femoral necks, with AUC values for OSTA for the left and right femoral necks of 80.0% and 85.8%, respectively. The corresponding figures for OSTC were 80.5% and 86.4%, respectively. For OSTA, sensitivity ranged from 72.1% to 74.6%, while specificity ranged from 73.9% to 81.4%; the corresponding values for OSTC were 73.2% to 73.4% and 74.8% to 82.6%, respectively.

**Table 3:** Predictive value of the OSTA and OSTC as compared to the BMD measurement in different anatomical sites.

Screening tool	Anatomical site	AUC (95% CI)	Optimal cut-off	Sensitivity, %	Specificity, %
OSTA	Lumbar vertebra L1	73.0 (69.6–76.5)	-1.93	73.4	62.7
OSTA	Lumbar vertebra L2	73.9 (70.5–77.3)	-1.89	73.7	62.9
OSTA	Lumbar vertebra L3	72.3 (68.8–75.8)	-1.93	70.2	64.0
OSTA	Lumbar vertebra L4	70.9 (67.3–74.5)	-2.93	58.4	74.9
OSTA	Lumbar vertebrae (L1–L4)	73.8 (70.4–77.2)	-2.93	59.4	77.4
OSTA	Left femoral neck	80.0 (76.7–83.3)	-2.93	72.1	73.9
OSTA	Right femoral neck	85.8 (81.7–90.0)	-3.99	74.6	81.4
OSTC	Lumbar vertebra L1	71.2 (67.7–74.8)	-25.2	64.3	69.2
OSTC	Lumbar vertebra L2	71.6 (68.0–75.1)	-25.1	63.8	69.4
OSTC	Lumbar vertebra L3	70.0 (66.5–73.7)	-18.7	73.9	56.0
OSTC	Lumbar vertebra L4	68.7 (65.0–72.4)	-30.9	54.9	73.4
OSTC	Lumbar vertebrae (L1–L4)	71.6 (68.1–75.1)	-23.3	68.1	64.7
OSTC	Left femoral neck	80.5 (77.2–83.7)	-30.8	73.4	74.8
OSTC	Right femoral neck	86.4 (82.5–90.2)	-40.1	73.2	82.6

AUC: Area under the curve; OSTA: Osteoporosis self-assessment tool for Asians; OSTC: Osteoporosis screening tool for Chinese.

The optimal cut-off points were determined based on Youden’s index.

## Discussion

Osteoporosis and its consequences are important issues that require strategies for early diagnosis and intervention at the community level. The percentages of osteoporosis following WHO criteria for the lumbar vertebrae (L1-L4) and left and right femoral necks were 46%, 29.2%, and 8.9%, respectively, and the average T-scores increased by age group. The percentage for lumbar vertebrae was higher than the percentage recorded in 2004 [32], while the percentages for the femoral necks matched the data reported in 2012 and 2015 [14,15]. Most osteoporotic cases progress asymptomatically; therefore, early detection is increasingly being seen as important in addressing the public health challenge. There is a consistent correlation between the risk of osteoporosis and population aging. Consequently, the prevention of fragility fractures among menopausal women has been a long-term national strategy, and cost-effectiveness must be taken into consideration to give high-risk people access to treatment. This issue demands the evolution of rapid diagnostic tools to enhance screening. Our study set out to evaluate the performance of OSTA and OSTC in Vietnam postmenopausal women.

DXA is regarded as the gold standard for detecting osteoporosis [29] and was used as a reference test in our study. The current versions of DXA use much lower radiation emissions than in the past, reducing as far as possible the risk from radioactive exposure [29]. However, DXA has limitations for screening due to its large size and lack of mobility. Further, using and interpreting DXA results require not only well-trained health workers but also suitable health facilities, which is a consistent restriction in the primary health system in Vietnam. If widely used as a screening tool, DXA could be an economic and human burden in LMICs [29], as over-reliance might lead to over-treatment and the possibility of overlooking BMD-independent osteoporosis through not including other risk factors related to lifestyle and fracture history. The prediction scores of both OSTA and OSTC were obtained using age and weight data and calculated easily without the need for complex methods. These two predictors can be collected simply in the community and have potential application in the routine practice of primary healthcare. Human bone mass is depleted with advancing age in both males and females. One common cause is age-related hormone disorder, especially in the postmenopausal period. Estrogen insufficiency promotes the risk of bone resorption when an imbalance occurs and disturbs the functioning of osteoclasts and osteoblasts [33]. This imbalance is not only related to age as an independent risk factor but also increases when combined with other risk factors such as diabetes, chronic obstructive pulmonary disease, hypertension, and glucocorticoid use. These factors are all involved in increasing the risk of osteoporosis and fractures later in life.

We found that the event risk was inversely proportional to BMI, which was in line with previous findings [34]. Risk scores measured at different skeletal sites show the same trend, with the T-scores decreasing with age and increasing with BMI. This correlation could be because the increase in BMI reflects a higher BMD, elevating the tool scores. In addition, postmenopausal estrogen replacement provokes an increase in high-fat mass, increasing BMI and subsequently increasing the tool scores [34]. Our T-scores were lower than in the findings of Yang et al. [35] at skeletal sites, showing a greater bone mass reduction in Vietnam participants.

The groups with the high-risk scores (OSTA score < -1, OSTC score ≤ 0) were predominant in our samples, which is consistent with previous studies [16,24,35]. The tool-based prevalence in this study was notably higher than that of Subramaniam et al. [36] in a sample with quite similar characteristics, namely Malaysians over the age of 39. The number of high-risk groups was central to our findings and three times greater than found in [36]. This distribution was clearly seen at all sites and aligns with one retrospective study carried out in Thailand [24].

To investigate the accuracy of OSTA and OSTC in osteoporosis prediction, WHO criteria were applied as the reference standard for the diagnosis of osteoporosis at anatomical sites. While OSTA has been widely assessed in Asia [16,32,35,37–41], evidence for OSTC is still limited. In our study, the validity of two tools was assessed for different sites, with the results obtained for femoral necks being better than those for lumbar vertebrae. In general, the predictive value was similar between the two tools. For OSTA, the AUC values for the lumbar vertebrae varied from 70.9% to 73.9%, which is similar to those of Fan et al. [39] and higher than those of Tang et al. [16]. Although the sensitivity of OSTA with the Vietnamese sample (58.4%– 73.7%) varied more widely than the Chinese sample (65%) [16], specificity was higher (62.7%– 77.4% vs. 48.9%) [16].

Another self-assessment tool, FRAX, which was published by Kanis in 2010 [19], has a declared accuracy equivalent to OSTA in the present study for the detection of osteoporosis. However, for femoral necks, OSTA performs better, with AUC values ranging from 80.0% to 85.8% [16,35,41], and is even comparable with other tools that use more complicated modeling [42]. OSTC also revealed good results for the lumbar vertebrae, with minimum and maximum AUC values of 68.7% and 71.6%, while recording better results for femoral necks with AUC values from 80.5% to 86.4%. OSTC values are even better than the findings of Jie-Long Tang [16] when different anatomical sites are taken into account.

Our findings strongly suggest that OSTA and OSTC are promising self-assessment tools for the early prediction of osteoporosis in Vietnamese postmenopausal women. Given their simplicity, the absence of complicated calculations, and their development specifically for Asian populations, the cost of OSTA and OSTC integration could be minimal while still achieving a good detection rate. These advantages strengthen the potential integration of the tools into community-based screening among LMICs. These tools could be very helpful for healthcare workers in primary care facilities, allowing them to quickly calculate and interpret the risk scores using any available calculation software.

While there should be potential for cost savings, there is a cost incurred in evaluating the positive results detected by the self-assessment tools using other diagnostic imaging techniques. These costs need to be considered to understand to what extent the tools enable cost savings and community-based implementation. The following strategy could be to optimize the diagnostic evaluation. First, high-risk subjects should be prioritized for positive evaluation, including people who have OSTA scores < -4 (high risk), OSTA scores between -4 and -2 (upper medium risk), or very low OSTC scores. Second, accessible, low-cost techniques, such as bone ultrasound, should be prioritized. People who are positive on bone ultrasound can then be examined by DXA to confirm the diagnosis. Third, clinical evaluation should be included for people at risk of osteoporosis according to OSTC because the tool output does not show any risk stratification. People who are at lower risk (OSTA scores ≥ -2, OSTC scores closing to 0) should participate in osteoporosis prevention programs and be assessed monthly by other, low-cost diagnostic imaging techniques. Finally, the government and non-profit organizations should financially support and scale-up prevention programs which could reduce the financial burden to individuals.

There are limitations to our study that need to be taken into account. First, our study used data from one hospital in a very large city in Vietnam; the results may not be generalizable for all postmenopausal women in other areas. Second, due to the limited data, we did not assess the performance of the two tools across different groups with a high risk of osteoporosis, such as people with non-communicable diseases (e.g., type 2 diabetes mellitus), lifestyle habits (i.e., smoking, alcohol assumption), and pharmaceutical treatments that affect the loss of bone mass and risk of osteoporosis [1]. Further studies should be conducted to evaluate the validity of OSTA and OSTC in different settings and in participants with diverse osteoporosis risk factors.

## Conclusions

OSTA and OSTC were shown to be useful self-assessment tools for osteoporosis detection in Vietnam postmenopausal women, with the predictive values ranging from medium to good accuracy. The tools are expected to benefit mass osteoporosis screening as part of a community-based strategy or in settings with limited resources. Further research is encouraged to determine the applicability of the tools for other populations and settings.

## Supporting information

S1 Table Comparison between risk of osteoporosis determined by OSTA and OSTC and the BMD classification in different anatomical sites. (DOCX)

S1 File Data and data dictionary. (RAR)
