# Supplementary material for: Evaluation of community-based screening tools for the early screening of osteoporosis in postmenopausal Vietnamese women
Source: PLoS One. 2022 Apr 5;17(4):e0266452. doi: 10.1371/journal.pone.0266452 (PMC8982873; doi:10.1371/journal.pone.0266452)
Supplement: S1 Table — (DOCX) [file pone.0266452.s001.docx]

**S1 Table: Comparison between risk of osteoporosis determined by OSTA and OSTC and the BMD classification in different anatomical sites**

| **Screening classification** | **T-score ≤ -2.5** | **2.5 < T-score < -1** | **T-score ≥ -1** |
| --- | --- | --- | --- |
|  | **Lumbar vertebra L1, n (%)** | | |
| **OSTA** |  |  |  |
| Low risk | 58 (15.1) | 173 (42.1) | 0 (0.0) |
| Medium risk | 197 (51.3) | 194 (47.2) | 2 (100.0) |
| High risk | 129 (33.6) | 44 (10.7) | 0 (0.0) |
| **OSTC** |  |  |  |
| No risk | 4 (1.0) | 41 (10.0) | 0 (0.0) |
| At risk | 380 (99.0) | 370 (90.0) | 2 (100.0) |
|  | **Lumbar vertebra L2, n (%)** | | |
| **OSTA** |  |  |  |
| Low risk | 58 (14.7) | 169 (42.6) | 4 (80.0) |
| Medium risk | 204 (51.6) | 189 (47.6) | 0 (0.0) |
| High risk | 133 (33.7) | 39 (9.8) | 1 (20.0) |
| **OSTC** |  |  |  |
| No risk | 7 (1.8) | 37 (9.3) | 1 (20.0) |
| At risk | 388 (98.2) | 360 (90.7) | 4 (80.0) |
|  | **Lumbar vertebra L3, n (%)** | | |
| **OSTA** |  |  |  |
| Low risk | 71 (16.3) | 154 (43.8) | 6 (66.7) |
| Medium risk | 226 (51.8) | 165 (46.9) | 2 (22.2) |
| High risk | 139 (31.9) | 33 (9.4) | 1 (11.1) |
| **OSTC** |  |  |  |
| No risk | 9 (2.1) | 34 (9.7) | 2 (22.2) |
| At risk | 427 (97.9) | 318 (90.3) | 7 (77.8) |
|  | **Lumbar vertebra L4, n (%)** | | |
| **OSTA** |  |  |  |
| Low risk | 54 (15.6) | 170 (38.7) | 7 (58.3) |
| Medium risk | 168 (48.6) | 222 (50.6) | 3 (25.0) |
| High risk | 124 (35.8) | 47 (10.7) | 2 (16.7) |
| **OSTC** |  |  |  |
| No risk | 8 (2.3) | 34 (7.7) | 3 (25.0) |
| At risk | 338 (97.7) | 405 (92.3) | 9 (75.0) |
|  | **Lumbar vertebrae (L1–L4), n (%)** | | |
| **OSTA** |  |  |  |
| Low risk | 53 (14.4) | 174 (40.9) | 4 (80.0) |
| Medium risk | 183 (49.9) | 209 (49.2) | 1 (20.0) |
| High risk | 131 (35.7) | 42 (9.9) | 0 (0.0) |
| **OSTC** |  |  |  |
| No risk | 5 (1.4) | 38 (8.9) | 2 (40.0) |
| At risk | 362 (98.6) | 387 (91.1) | 3 (60.0) |
|  | **Left femoral neck, n (%)** | | |
| **OSTA** |  |  |  |
| Low risk | 15 (6.4) | 212 (38.0) | 4 (66.7) |
| Medium risk | 107 (45.9) | 284 (50.9) | 2 (33.3) |
| High risk | 111 (47.6) | 62 (11.1) | 0 (0.0) |
| **OSTC** |  |  |  |
| No risk | 1 (0.4) | 42 (7.5) | 2 (33.3) |
| At risk | 232 (99.6) | 516 (92.5) | 4 (66.7) |
|  | **Right femoral neck, n (%)** | | |
| **OSTA** |  |  |  |
| Low risk | 0 (0.0) | 220 (30.9) | 11 (73.3) |
| Medium risk | 20 (28.2) | 369 (51.9) | 4 (26.7) |
| High risk | 51 (71.8) | 122 (17.2) | 0 (0.0) |
| **OSTC** |  |  |  |
| No risk | 0 (0.0) | 41 (5.8) | 4 (26.7) |
| At risk | 71 (100.0) | 670 (94.2) | 11 (73.3) |
